# Photoluminescence Performance and Photocatalytic Activity of Modified Carbon Quantum Dots Derived from Pluronic F127

**DOI:** 10.3390/polym15040850

**Published:** 2023-02-08

**Authors:** Linlin Liu, Yue Zhang, Youliang Cheng, Jing Chen, Fengjuan Li

**Affiliations:** 1 Faculty of Printing, Packaging Engineering and Digital Media Technology, Xi’an University of Technology, Xi’an 710048, China; 2 School of Mechanical and Electrical Engineering, Xinjiang Institute of Technology, Aksu 843000, China

**Keywords:** carbon quantum dots, hydrothermal method, photocatalytic activity

## Abstract

The photocatalytic degradation of organic dyes in waste water using carbon quantum dots (CQDs) remains a hot topic due to the importance of environmental protection. However, identifying suitable carbon resources and successful surface modification are still challenging. Herein, the hydrothermal method and surface modification of ammonia and thionyl chloride were applied to synthesize CQDs with different surface groups using PEO_106_PPO_70_PEO_106_ (Pluronic F127) as a carbon source. The average particle size of the as-prepared CQDs was in the range of 2.3–3.5 nm. The unmodified CQDs had the highest relative photoluminescence intensity, while all as-prepared CQDs exhibited abnormal photoluminescence located outside the scope of the visible spectrum. Interestingly, CQDs modified with ammonia achieved a degradation rate of 99.13% (15 d) for 50 mg/L indigo carmine solution, while CQDs modified with thionyl chloride reached a degradation rate of 97.59% (15 d) for light green SF yellowish solution. Therefore, in this work, two typical organic dyes can be effectively photocatalytically degraded by as-prepared CQDs, with suitable surface modification.

## 1. Introduction

Recently, many organic dyes remaining in wastewater from printing and dyeing industries, which are difficult to degrade naturally, have led to serious environmental pollution and human health problems [1,2]. However, traditional methods for treating organic pollutants, such as adsorption [3,4,5], flocculation [6], ion exchange, membrane separation, electrolysis [7], and activated sludge [8], are generally limited by high cost, high environmental impact, and harmful by-products. These limitations make the efficient application of traditional methods unfeasible [9]. Photocatalytic technology has been widely used for organic dye degradation due to its efficient, economical, and sustainable advantages [10]. Nevertheless, problems due to the high recoupling rates of photogenerated carriers (e.g., electrons and holes) and the inefficient utilization of sunlight in some photocatalytic devices remain unsolved. Fortunately, carbon quantum dots (CQDs) manifest the potential to address these issues [11]. Currently, the application of CQDs in the photocatalytic degradation of organic dyes is drawing the attention of many researchers [12,13].

In 2004, Xu et al. [14] first discovered CQDs during the process of the preparation of single-walled carbon nanotubes using the purification arc. To our knowledge, CQDs are novel nanoparticles with abundant functional groups on their surfaces, showing unique photoluminescence (PL) properties [15]. They possess the advantages of simple synthesis, low cost, low toxicity, and biological compatibility [12,16,17], which have been widely applied in the fields of anti-counterfeiting [18], biology and fluorescence image [19,20], biosensing, and photocatalysis [21]. The synthesis route of CQDs mainly includes “top-down” and “bottom-up” strategies [22]. The former obtains CQDs from carbon materials via arc discharge [22], laser ablation [23], and electrochemical methods [24]. The latter obtains CQDs from carbon precursors, such as glucose and fruits, via the hydrothermal method [25], ultrasonic method [26], and microwave irradiation [22]. Particularly, the hydrothermal method is the most effective method for the synthesis and modification of CQDs because of the simple synthesis process, low cost, controllable morphology, and high yield [15,27].

Relevant works have been conducted on the preparation of CQDs by the hydrothermal method and the photocatalytic degradation of organic dyes. Remli et al. [28] used watermelon rinds as carbon-containing precursors to fabricate CQDs using the hydrothermal method. The degradation rate was 68.9% using the above CQDs as the photocatalyst when methyl orange (MO) dye was irradiated under an artificial source for 2 h. Najjar et al. [29] synthesized CQDs with impressive PL intensity and quantum yield (QY) via the hydrothermal method using cordia myxa L. as the carbon precursor. The obtained CQDs were used for the photocatalytic degradation of methylene blue (MB), crystal violet (CV), methyl orange (MO), and Eriochrome Black T (EBT) dyes under UV irradiation, and these results suggested that they could completely degrade these dyes within 1 h under UV irradiation. Dejpasand et al. [30] prepared N-doped CQDs (NCQDs) using citric acid and urea as raw materials by the hydrothermal method. The obtained NCQDs can degrade MB dye up to 98% after 150 min under visible light irradiation. For improving the photocatalytic degradation efficiency of organic dyes, the electronic structure of CQDs can be changed by elemental doping or surface modification to increase the electron conversion intensity in CQDs [11,27]. Hui et al. [15] prepared heteroatom doped CQDs from rice husk by the hydrothermal method, and the N and Bi doped CQDs exhibited degradation rates of 72.16% and 68.91% for MB. Rani et al. [31] used empty fruit bunches as a raw material to produce N-doped CQDs by the hydrothermal method, which demonstrated about a 97% and 98% degradation rate for MB and malachite green within 3 h and 2 h under sunlight irradiation, respectively. Additionally, there are few reports related to the preparation of CQDs from block polyether surfactants for the photocatalytic degradation of organic dyes.

For the photocatalytic degradation of organic dyes, the reported research has studied mainly common organic dyes, such as MB and MO, but there are other organic dyes as well. Indigo carmine (IC) dyes, the oldest anionic indigo dyes, are widely applied in the textile, food, and cosmetic industries [32]. IC dyes can cause permanent damage to the human body by irritating the eyes and skin. Moreover, IC is carcinogenic and can pose a serious threat to human health [33]. Light green SF yellowish (LG SF), a triarylmethane dye [34], will pollute water and cause harm to aquatic organisms when dissolved in water, with the potential to accumulate and permeate skin. Thus, LG SF and its metabolites have certain induced carcinogenic effects on living systems [35]. Therefore, the effective removal of IC and LG SF dyes is an urgent problem to be solved. In this regard, we consider the effective photocatalytic degradation of both IC and LG SF dyes by using CQDs.

**Figure 1:**
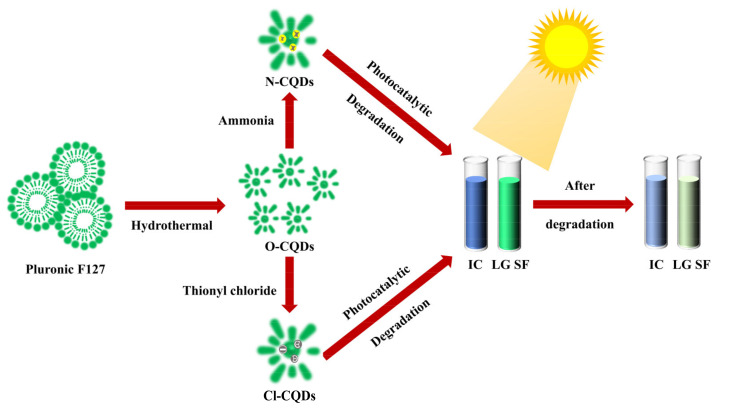
Synthesis of CQDs and organic dye degradation processes (Pluronic F127: PEO_106_PPO_70_PEO_106_; CQDs: carbon quantum dots; IC: indigo carmine; LG SF: light green SF yellowish).

Herein, PEO_106_PPO_70_PEO_106_ (Pluronic F127) was used as the carbon source, and the CQDs were synthesized by the hydrothermal method. Then, the CQDs were modified by using ammonia and thionyl chloride. The morphology, photoluminescence, and photocatalytic degradation performance of the as-prepared CQDs were investigated. The unmodified CQDs had the highest relative fluorescence intensity, while the modified CQDs can effectively degrade indigo IC and LG SF. Figure 1 displays the preparation of CQDs and the degradation process of IC and LG SF.

## 2. Materials and Methods

### 2.1. Materials

Pluronic F127 and ammonia were purchased from Tianjin Tianli Chemical Reagent Co., Ltd. (Tianjin, China). Sulfoxide chloride was purchased from Shanghai Aladdin Biochemical Technology Co., Ltd. (Shanghai; China) LG SF and IC were provided by Shanghai Lanji Biological Co., Ltd. (Shanghai; China). 

### 2.2. Synthesis of CQDs

Typically, 0.469 g of Pluronic F127 was added to 100 mL of deionized water and stirred for 30 min. Next, 75 mL of the above solution was poured into a 100 mL autoclave and held for 24 h in an oven at 120 °C. When the process was completed, the system was naturally cooled to room temperature. Afterwards, we centrifuged the product at 10,000 rpm for 10 min. Finally, supernatants were purified using a dialysis bag (3000 Da) and denoted as O-CQDs.

### 2.3. Surface Modification of CQDs

Typically, 15 mL of O-CQDs solution was mixed with 804 μL of 25% ammonia solution and thionyl chloride, respectively. The mixtures were stirred for 10 min. Next, the above mixed solution was shifted to a 20 mL reactor and held at 100 °C for 5 h. After cooled to room temperature, the products were centrifuged at 10,000 rpm for 10 min. Finally, the supernatants were purified using a dialysis bag (3000 Da) and denoted as N-CQDs and Cl-CQDs, respectively.

### 2.4. Photocatalytic Degradation Assessment of IC and LF SF

A total of 5 mL of as-prepared Cl-CQDs and N-CQDs solutions were dispersed in 5 mL of 50 mg/L LG SF and IC solutions, respectively. Then, the mixture was placed in a dark environment for 2 h to reach the equilibrium of adsorption and desorption. Finally, the mixtures were irradiated under natural light (with sunlight as the light source) for 2 h. After prescribed time intervals, the concentration of organic dyes was determined by UV–Vis absorption spectroscopy. As a control, 5 mL deionized water was added to the organic dye solution under the same conditions. For investigating the reuse of N-CQDs and Cl-CQDs, the photocatalytically degraded IC and LG SF solutions were centrifuged at 22,000 rpm for 20 min to collect N-CQDs and Cl-CQDs. Then, the isolated products were added to the undegraded IC and LG SF dye solutions, respectively, for secondary degradation.

The photocatalytic degradation rate of the CQDs in IC and LG SF can be calculated by the following equations [32]:

Degradation = (1−C/C_0_) × 100% (1)

where C_0_ is the starting concentration of the dye solution, and C is the concentration of the dye solution at degradation time t. The concentration of the dye was evaluated by using the Beer–Lambert law (in Equation (2)):

A = Cεl(2)

where A is the absorbance of the dye solution, C is the concentration of the dye solution, ε is the molar absorption coefficient, and l is the length of the optical path.

### 2.5. Characterization

The morphologies of the CQDs were investigated by transmission electron microscopy (TEM, JEM-2100 plus, Tokyo, Japan) and high-resolution transmission electron microscopy (HRTEM, JEM-3010, Tokyo, Japan). Sample solutions were dropped onto a Cu grid and then dried at room temperature for TEM testing. A UV-Vis spectrophotometer (Shimadzu UV-2550, Kyoto, Japan) was applied to measure the UV-Vis absorption spectra of the as-prepared samples. A fluorescence spectrometer (Hitachi-4600, Tokyo, Japan) was used to measure the PL spectra of the samples.

## 3. Results and Discussion

### 3.1. Morphology of CQDs

**Figure 2:**
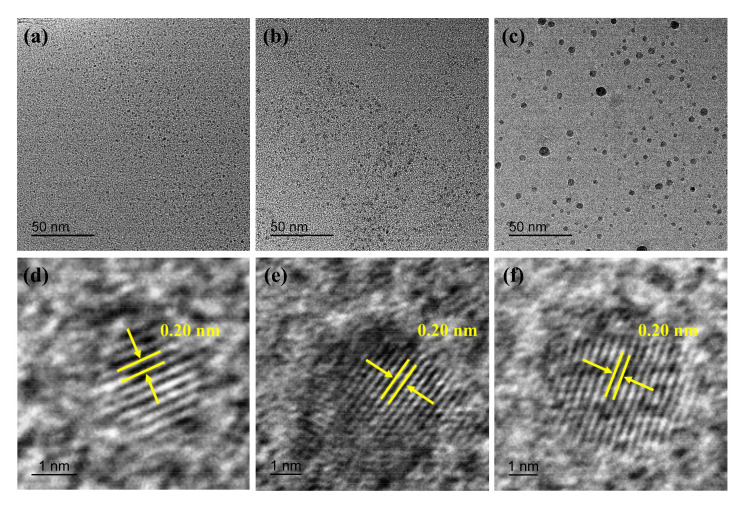
TEM images of CQDs: (**a**) O-CQDs, (**b**) N-CQDs, (**c**) Cl-CQDs; and the lattice spacing of CQDs in HRTEM images: (**d**) O-CQDs, (**e**) N-CQDs, (**f**) Cl-CQDs.

Figure 2 shows the TEM images and the lattice spacing of the O-CQDs, N-CQDs, and Cl-CQDs. The CQDs fabricated by the hydrothermal method in this work exhibit a spherical shape, with a small particle size (in Figure 2a–c). According to HRTEM images (shown in Figure 2d–f), we can observe that the lattice spacing of the three samples is about 0.20 nm, which is similar to the (100) plane of graphite verifying the formation of CQDs [36,37]. In addition, the HRTEM images reveal that the crystals are often accompanied by some defects. Most of the defects in the products are generated at high temperatures due to the interactions of groups on Pluronic F127, yet they also favor the formation of CQDs.

**Figure 3:**
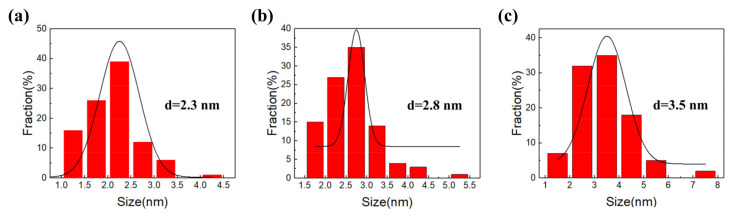
The size distribution graphics of CQDs (**a**) O-CQDs, (**b**) N-CQDs, (**c**) Cl-CQDs.

Nano Measure software was used to calculate the diameter statistics of the CQD nanoparticles in Figure 2a–c, and Origin software was utilized to plot the particle size statistics as a histogram and fit them with Gaussian curves. Figure 3 reveals the size distribution of O-CQDs, N-CQDs and Cl-CQDs. We find that the average particle size of O-CQDs, N-CQDs, and Cl-CQDs is about 2.3 nm, 2.8 nm, and 3.5 nm, respectively. Furthermore, it was observed that the average diameter of the modified CQDs was higher than that of the unmodified CQDs, which was similar to the results in our previous work [12]. This result might be attributed to the fact that the surface of the modified CQDs possesses N- or Cl- groups, with the exception of the O-containing groups.

### 3.2. Optical Performance Changes of CQDs with Different Functional Groups

**Figure 4:**
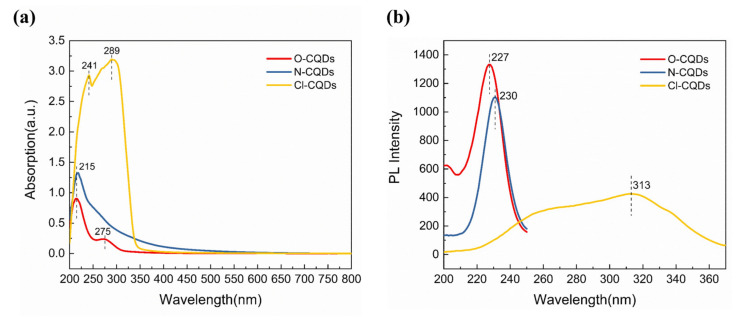
(**a**) UV-Vis absorption spectra and (**b**) PL spectra of O-CQDs, N-CQDs, and Cl-CQDs.

The UV-Vis absorption spectra of the samples are displayed in Figure 4a. To our knowledge, the absorption peaks caused by the π-π* transition (C=C) and the n-π* transition (C=O) were located in the range of 200–300 nm for all samples [26,38,39,40]. Figure 4b shows the PL emission peaks for samples of O-CQDs, N-CQDs, and Cl-CQDs at different excitation wavelengths (280 nm, 280 nm, and 380 nm, respectively). At an excitation wavelength of 280 nm, O-CQDs and N-CQDs exhibit emission peaks at 227 nm and 230 nm, respectively. These emission peaks match the absorption bands of O-CQDs and N-CQDs at about 215 nm. When the excitation wavelength was 380 nm, the emission peak for Cl-CQDs appeared at 313 nm, and the emission peak matches the absorption band of Cl-CQDs at 289 nm [41]. Compared with the N-CQDs and Cl-CQDs, the sample of O-CQDs has the highest relative PL emission intensity. This result is probably attributed to the different internal structures of the carbon sources [12]. According to the PL spectra of O-CQDs, N-CQDs and Cl-CQDs, they exhibit special optical performances similar to up-conversion luminescence, despite that these emission peaks locate outside the scope of the visible spectrum. These abnormal phenomena may be caused by the special structure and components of the as-prepared CQDs derived from Pluronic F127 in this work, which will be further investigated in future work.

### 3.3. Photocatalytic Activity of As-Prepared CQDs

**Figure 5:**
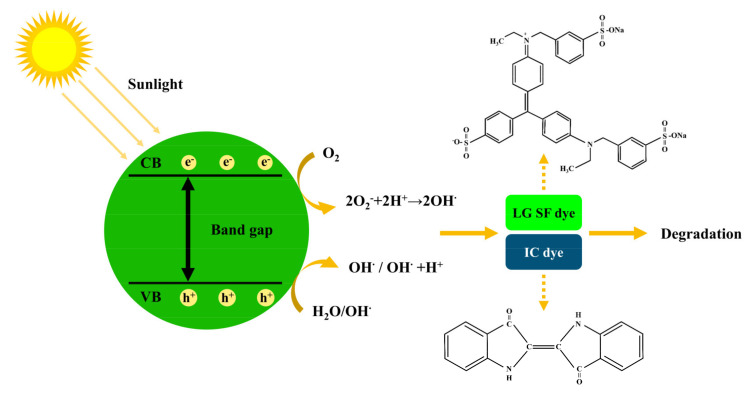
Schematic diagram of the degradation mechanism of CQDs on IC and LG SF dyes. (CB: Conduction Band; VB: Valence Band).

To our knowledge, photocatalytic reactions are triggered by the production of the excess electrons (e^-^) and holes (h^+^). Typically, the photocatalytic reaction of CQDs undergoes three steps. Firstly, when irradiated under suitable light, CQDs will adsorb photons with energy hν, and electrons will jump from the ground state to the excited state (in Equation (3)), consequently producing e^-^ and h^+^ pairs. Secondly, •OH and •O_2_^−1^ formation causes the separation of e^-^/h^+^ pairs, which transfer to the surface of the CQDs. Finally, •OH will act as an initiator for the photocatalytic degradation of organic dyes on the surface of CQDs [11,12]. Figure 5 gives the mechanism for the photocatalytic degradation of IC and LG SF dyes by CQDs.

CQDs + hν → e^−^+ h^+^ (3)

**Figure 6:**
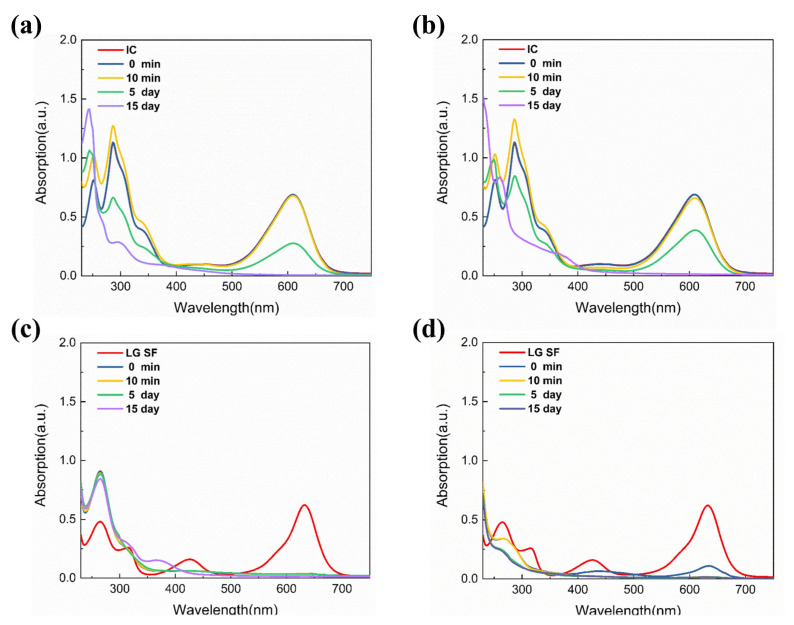
UV-Vis absorption spectra of IC solution under natural light for different time periods after CQDs were added: (**a**) N-CQDs, (**b**) Cl-CQDs; and UV-Vis absorption spectra of LG SF solution under natural light for different time periods after CQDs were added: (**c**) N-CQDs, (**d**) Cl-CQDs.

According to our previous report [7], CQDs derived from aqua mesophase pitch can be modified by ammonia and thionyl chloride, and they exhibited high photocatalytic activity in the degradation of MB, IC, and Rhodamine B. Thus, in this work, we will focus on the photocatalytic degradation of typical dyes by using surface modified CQDs derived from Pluronic F127. Figure 6 gives the UV-Vis absorption spectra of IC and LG SF solutions under natural light for different time periods after the addition of N-CQDs and Cl-CQDs, respectively. According to Figure 6a,b, the UV-Vis absorption spectra for IC solution at 0 min after adding N-CQDs and Cl-CQDs were basically the same as those for the original IC solution, and they changed noticeably between 480 nm and 700 nm after 10 min. The relative intensity of the absorption peak for IC solution decreases significantly 10 min after adding CQDs, demonstrating that the addition of CQDs may cause a significant change in IC molecule structure, subsequently effectively degrading the IC dye [12]. In addition, the degradation rate of IC dye using N-CQDs and Cl-CQDs increases with the prolongation of the irradiation time with natural light.

Additionally, after adding Cl-CQDs and N-CQDs, the relative intensity of UV-Vis absorption peak for the LG SF solution was significantly weakened in the visible region, especially at 632 nm. This result means that Cl-CQDs and N-CQDs have a positive photocatalytic degradation effect on LG SF in the visible region (shown in Figure 6c,d). During the degradation process, the trimethylbenzene component undergoes deethylation, which allows for the removal of a benzene ring and the formation of a diphenylmethane structure [42]. Then, the gradual opening and/or removal of the benzene ring will form small molecules by inducing the ring opening and/or branched chain oxidation. These results illustrate that the CQD surface groups are responsible for different degradation effects and degradation rates of IC and LG SF dyes in the solutions.

**Figure 7:**
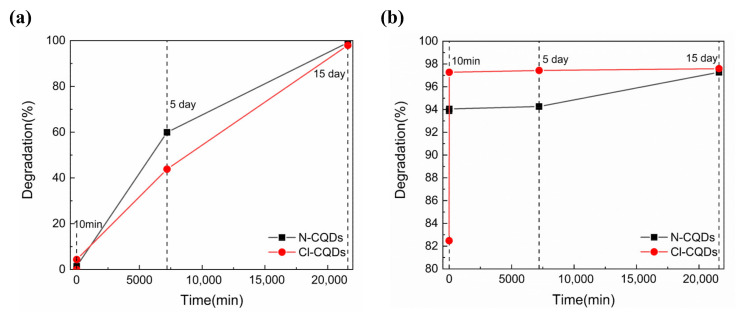
The photocatalytic degradation efficiency of N-CQDs and Cl-CQDs derived from Pluronic F127 for (**a**) IC and (**b**) LG SF dyes.

Figure 7 depicts the photocatalytic degradation efficiency of IC and LG SF dyes by using the N-CQDs and Cl-CQDs obtained in this work. Overall, the degradation rate of IC and LG SF dyes using using N-CQDs and Cl-CQDs increases with the prolonging of the degradation time. For the degradation of IC solutions, the highest degradation rate was obtained after 15 d for both N-CQDs (99.13%) and Cl-CQDs (97.97%). However, the degradation rate of IC dyes by using N-CQDs and Cl-CQDs is less than 10% when the degradation time is 10 min. When the degradation time is extended to 5 d, the degradation rate of the IC dyes is 59.94% and 43.83%, respectively. Therefore, it is relatively more effective to use N-CQDs for the degradation of IC solutions compared with Cl-CQDs (shown in Figure 7a). As shown in Figure 7b, it can be seen that the degradation rate of LG SF dyes by using N-CQDs and Cl-CQDs immediately reaches 93.89% and 82.48%, once N-CQDs and Cl-CQDs are added to LG SF solution. After 10 min of degradation, the degradation rate of LG SF in the solution using Cl-CQDs reaches 97.27%. Thereafter, there is only a slight increase in the degradation rate with further prolongation of the degradation time. According to Figure 7b, it can be inferred that a relatively high degradation rate of LG SF in the solution using Cl-CQDs will be achieved after only 10 min. The degradation rate of LG SF in the solution using N-CQDs reached the greatest value of 97.27% when the degradation time was 15 d under natural light. During the whole degradation process, the degradation rate of LG SF in the solution using N-CQDs remains little changed, from 93.89% to 97.27%. Compared with Cl-CQDs, the degradation rate of LG SF in the solution using N-CQDs was lower after same degradation time. The photocatalytic degradation of IC and LG SF in the solutions using N-CQDs and Cl-CQDs centrifuged from degraded IC and LG SF solutions was carried out for the second time. Then, the degradation rate of IC dyes by N-CQDs and Cl-CQDs after 15 d was about 96.46% and 95.58%, respectively. Moreover, the degradation rate of LG SF dyes by N-CQDs and Cl-CQDs after 15 d was about 93.74% and 93.25%, respectively. Thus, N-CQDs and Cl-CQDs retain a high degradation rate of IC and LG SF dyes in the solutions over the second cycle.

Table 1 demonstrates the degradation efficiency of different catalysts for both IC and LG SF dyes. For the photocatalytic degradation of IC dyes, our current strategy can reach a very high degradation rate, although it requires more time, which may due to the inconsistencies in pH, initial dye concentration, and light intensity [43]. For the photocatalytic degradation of LG SF dyes, fewer studies are available. Compared with the established literature, it is evident that the surface-modified CQDs can degrade LG SF dyes with a relative high degradation rate in a short period of time. These results suggest that surface-modified CQDs provide a new pathway for the degradation of organic dyes.

Furthermore, Equation (4) was used to investigate the reaction kinetics of the degradation of IC and LG SF in the solutions by N-CQDs and Cl-CQDs, according to the Langmuir–Hinshelwood (L-H) kinetic model [46,47]:

In(C_0_/C) = kt (4)

where C_0_ is the starting concentration of the dye solution, C is the dye concentration at t time, and k is the reaction rate constant.

**Figure 8:**
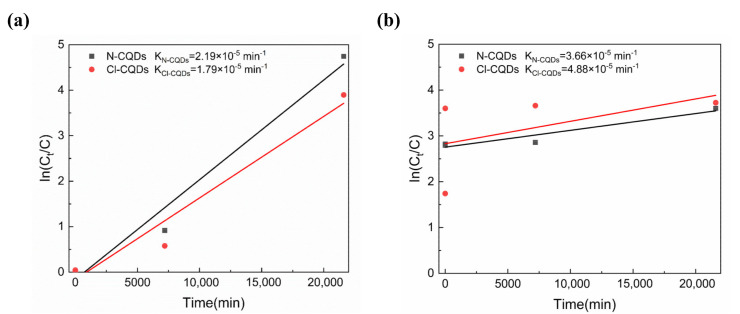
Diagrams of In(C_0_/C) versus time (t) for the degradation of (**a**) IC and (**b**) LG SF dyes in the solutions by using N-CQDs and Cl-CQDs.

Figure 8 exhibits the In(C_0_/C) versus time (t) for the degradation of IC and LG SF dyes in the solutions by using N-CQDs and Cl-CQDs. According to Figure 8, there is a linear relationship between In(C_0_/C) and t for the degradation of IC and LG SF dyes, meaning that the photocatalytic degradation of IC and LG SF dyes in the solutions by N-CQDs and Cl-CQDs is in accordance with the first-order kinetics. For the IC solutions, the degradation rate constants by using N-CQDs and Cl-CQDs are 2.19 × 10^−5^/min and 1.79 × 10^−5^/min, respectively, implying that N-CQDs can exert a higher photocatalytic effect. As for LG SF solutions, N-CQDs and Cl-CQDs display the degradation rate constants of 3.66 × 10^−5^/min and 4.88 × 10^−5^/min, respectively, suggesting that the photocatalytic degradation of LG SF dyes in the solution by Cl-CQDs is superior.

## 4. Conclusions

In this work, we synthesized CQDs with different surface groups via the hydrothermal method and subsequent modification using Pluronic F127 as the carbon source. The average diameter of the CQDs prepared at a hydrothermal temperature of 120 °C for 24 h was 2.3 nm, and the average diameter of the CQDs modified by ammonia and thionyl chloride was 2.8 nm and 3.5 nm, respectively. The unmodified CQDs showed the highest relative PL intensity. In addition, the as-prepared CQDs from Pluronic F127 exhibited abnormal PL characteristics. The photocatalytic degradation of IC and LG SF dyes in the solutions of CQDs modified with ammonia and thionyl chloride was in accordance with the first-order kinetics. The degradation rate of IC dyes in the solution of CQDs modified with ammonia achieved 99.13% after 15 d, while that of LG SF dyes in the solution of CQDs modified with thionyl chloride was 97.59% after 15 d. These results suggest that CQDs with different surface groups and structures play different roles in photoluminescence and photocatalytic degradation performance. Consequently, this work not only provides a new route for the preparation of different CQDs, but also exhibits a potential application for the photoluminescence and photocatalytic degradation of materials.

## Figures and Tables

**Table 1:** Photocatalytic degradation efficiency of different photocatalysts for IC and LG SF dyes.

Photocatalyst	Organic Dyes	Light Source	Concentration of Dyes	Degradation Efficiency	Reaction Time	Reference
graphene oxide (10%)–nano-titania	IC	Visible light	10 mg/L	98%	60 min	[44]
Fe (2 mol%) doped TiO_2_	IC	100 W fluorescent bulb	10 mg/L	94.76%	60 min	[32]
Nd (2%)-TiO_2_-GO (graphene oxide)	IC	Simulated solar light	20 mg/L	75%	30 min	[45]
Bi_5_Ti_3_FeO_15_	IC	Sunlight	30 mg/L	13% (pH = 8)	240 min	[43]
N-CQDs	IC	Visible light	30 mg/L	97%	240 min	[12]
N-CQDs	IC	Natural light	50 mg/L	99.13%	15 day	This work
Cl-CQDs	IC	Natural light	50 mg/L	97.97%	15 day	This work
UV/IO_3_^−^	LG SF	Low-pressure mercury lamp (15 mW cm^−2^)	10 mg/L	79% (0.5 mM IO_3_^−^)	10 min	[35]
				93% (1 mM IO_3_^−^)		
				98% (10 mM IO_3_^−^)		
			20 mg/L	43% (0.5 mM IO_3_^−^)		
				60% (1 mM IO_3_^−^)		
				85% (10 mM IO_3_^−^)		
N-CQDs	LG SF	Natural light	50 mg/L	94.05%	10 min	This work
Cl-CQDs	LG SF	Natural light	50 mg/L	97.27%	10 min	This work

## Data Availability

Not applicable.
